# Transplant or no transplant for *TP53* mutated AML

**DOI:** 10.18632/oncotarget.28652

**Published:** 2024-10-01

**Authors:** Talha Badar, Moazzam Shahzad, Ehab Atallah, Mark R. Litzow, Mohamed A. Kharfan-Dabaja

**Keywords:** AML, *TP53* mutation, allogeneic stem cell transplant

*TP53* mutations (mut) occurs in 10–15% of cases of acute myeloid leukemia (AML), more commonly associated with therapy-related AML (t-AML) and those harboring complex cytogenetics [1, 2]. *TP53*-mut AML is inherently resistant to conventional chemotherapies [3], and still has a dismal prognosis in the era of novel therapies, including venetoclax-based therapies.[4] Allogenic hematopoietic stem cell transplant (allo-HCT) remains a potentially curative option in *TP53*-mut AML, but only 10–15% of these patients are able to receive an allo-HCT.[5, 6] Factors known to be predictive of transplant outcomes include age, comorbidities, concurrent complex cytogenetics (CG), depth of response prior to transplant, conditioning intensity and post-transplant immune-mediated graft-versus-leukemia (GVL) effect [7–10]. In a recently conducted multicenter real-world study utilizing data from the multi-institutional Consortium of Myeloid Malignancies and Neoplastic Diseases (COMMAND), we evaluated outcomes of *TP53*-mut AML patients with evolving frontline therapies, a dismal survival of 8.5 months (range (R), 6.14–10.05) was found and was not significantly different with intensive, non-intensive or venetoclax-based induction therapies [6]. In this study while only 16% of patients were successfully bridged to allo-HCT, this was the only variable associated with improved survival in multivariable analysis.

**Figure 1:**
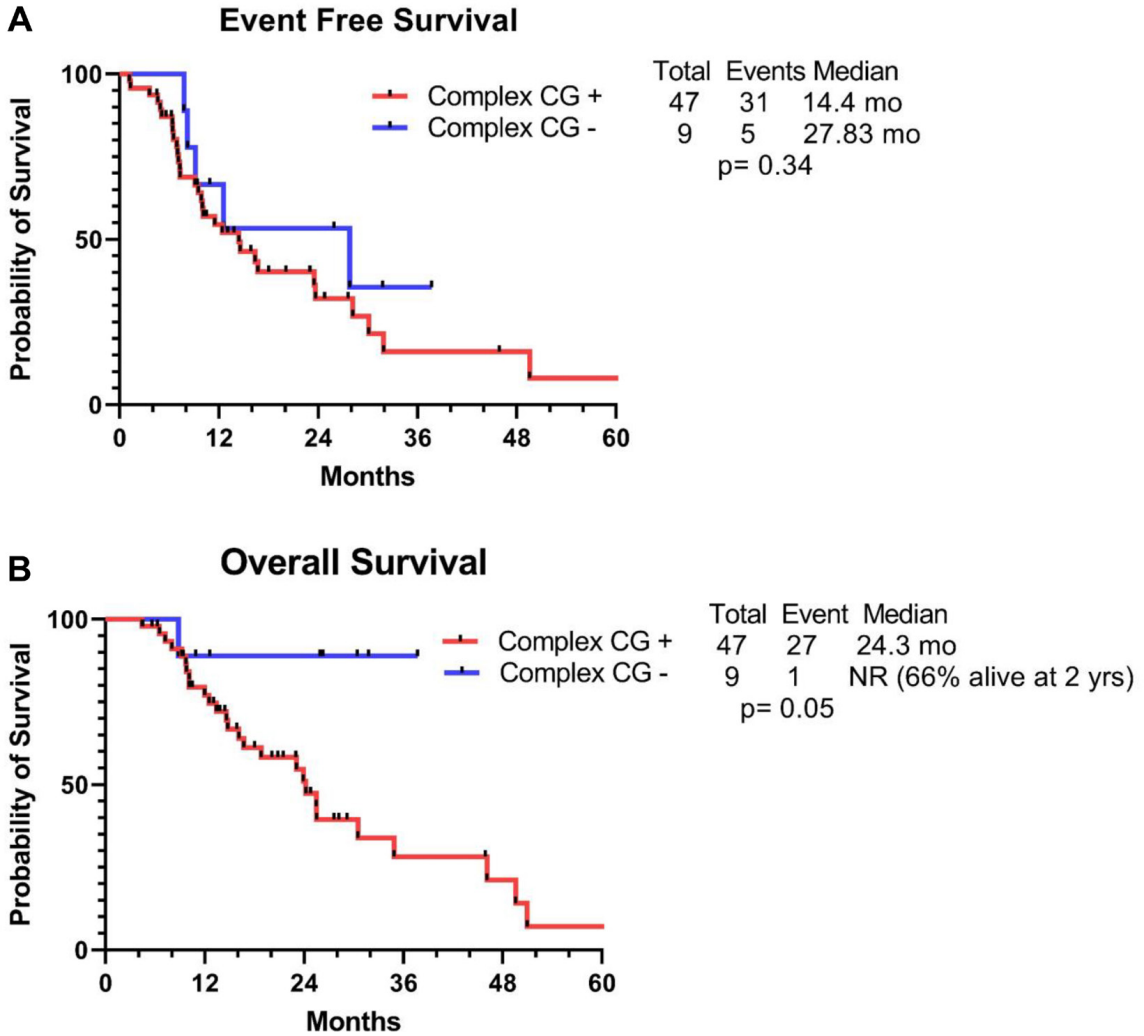
Transplant outcome with complex CG. Kaplan Meier survival curves demonstrating (**A**) Event-free survival and (**B**) Overall survival among *TP53*-mutated AML patients with and without complex cytogenetics.

Subsequently, we conducted an analysis to look for predictors of response among allografted patients with *TP53*-mut AML, utilizing data again from the COMMAND [5]. In this study, 370 patients with *TP53*-m AML who were treated between 2012 and 2021 were included, out of which 68 (18%) were bridged to an allo-HCT; 49 (78%) after first induction and 19 (22%) after salvage therapy. In this cohort of *TP53*-mut AML, majority of patients had complex CG (82%) and had multi-hit *TP53*-mut (66%). Fifty-seven percent of patients received reduced intensity conditioning (RIC) and 43% received myeloablative conditioning (MAC). Seventy-six percent of patients underwent allo-HCT in complete remission (CR) with or without count recovery (CRi), and among 38 patients evaluated for *TP53*-mut prior to allo-HCT, 29% (*n* = 11) had clearance of *TP53*-mut at the time of transplantation. The median event-free survival (EFS) was 12.4 months; 14.6 vs. 8.9 months (*p* = 0.19) for allo-HCT after first induction and after salvage therapy, respectively. And the median overall survival (OS) observed in this analysis was 24.5 months; 30.5 vs. 20.20 months (*p* = 0.01) for allo-HCT after first induction and after salvage therapy, respectively. In multivariate analysis, maintenance of CR/CRi day 100 post allo-HCT (EFS = HR, 0.24, 95% CI, 0.10–0.57/ OS = HR, 0.22, 95% CI: 0.10–0.50) and occurrence of chronic graft-versus-host disease (cGVHD) (EFS = HR, 0.21, 95% CI: 0.09–0.46/ OS = HR,0.34, 95% CI: 0.15–0.75), retained significance for improved survival.[5] In this study, we did not report the impact of complex CG on transplant outcome, hence we reviewed our data and conducted a survival analysis among allo-HCT recipients after first induction with complex CG. We observed inferior EFS (14.4 vs. 27.8 months, *p* = 0.34) and OS (24.3 vs. not reached (NR; 66% alive at 2 years), *p* = 0.05) among transplanted patients with and without complex CG, respectively (Figure 1). As noted, EFS was not statistically significant, probably due to small sample size.

The reported outcomes are in line with previous observations where better long-term outcomes were observed when allo-HCT was performed in CR1 [11]. The European Society for Blood and Marrow Transplantation (EBMT) evaluated outcomes of patients with *TP53*-mutated AML receiving allo-HCT in CR1 [9]. Patients with concurrent complex CG or loss of 17p had poorer outcomes, with 2-year leukemia-free survival of 15%, while patients with no evidence of complex CG or 17p loss had a 2-year OS comparable to that of patients with wild type TP53 [9]. Similarly, other studies have shown inferior outcomes with concurrent complex CG in *TP53-*mut AML [12, 13]. Our data also showed a trend towards inferior outcome with concurrent complex CG compared to *TP53*-mut AML without complex CG.

**Figure 2:**
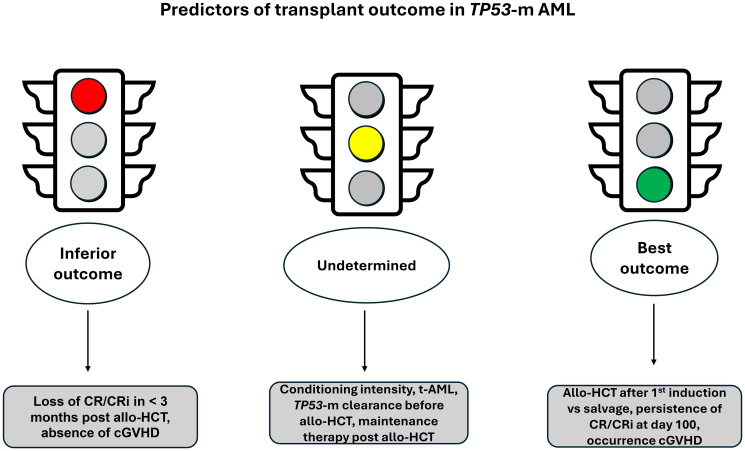
Diagrammatic illustration of factor that predicted transplant outcomes in *TP53*-mutated AML (Badar et al., Leukemia 2023).

In summary, this study reported improved survival when allo-HTC was performed in CR1 versus after later lines of therapy. Optimal benefit was observed in patients who maintained response at day 100 post allo-HCT and had cGVHD. Conditioning intensity, therapy-related AML (t-AML), *TP53*-mut clearance prior to allo-HCT and post allo-HCT maintenance therapy did not appear to significantly influence survival outcome (Figure 2). The strength of the study lies in its multi-institutional involvement representing a broad patient population and including treatment practices across different centers, thus reducing the bias of single-center studies. The study included comprehensively annotated molecular data to assess the influence of genetic aberrations on outcome, which is unique compared to other reported data on transplant outcomes in high-risk AML [8, 11].

Interestingly, in this study survival was not influenced by pre-transplant measurable residual disease (MRD) negativity by flow cytometry or clearance of *TP53*-mut clone. Recently, in a phase III randomized study (ASAP trial) outcomes with immediate allo-HSCT after sequential conditioning without attempt to induce a CR versus attempting to induce remission with intensive chemotherapy prior to allo-HSCT in patients with high-risk AML were evaluated [14]. The study reported that patients with sub-optimal response (less than CR) prior to allo-HCT did not benefit from additional intensive chemotherapy. Close surveillance and sequential conditioning followed by allo-HCT resulted in comparable OS. This strategy may be pursued in *TP53*-mut AML which is inherently resistant to conventional chemotherapy and would rely on anti-leukemia activity via donor allo-immune responses to improve survival.

We acknowledge the limitations of our retrospective analysis, including selection bias, heterogeneity of data from participating institutions and lack of complete molecular data prior to allo-HCT that might have influenced outcome. Yet, results are encouraging and show a benefit of allo-HCT in improving long-term outcome in this poor prognostic disease, where there is still a scarcity of effective therapies.
